# Evaluating pump-assisted larval transfer for scaling coral larval restoration interventions

**DOI:** 10.1371/journal.pone.0346728

**Published:** 2026-04-17

**Authors:** Chelsea Waters, Marine Gouezo, Peter L. Harrison, Christopher Doropoulos

**Affiliations:** 1 Faculty of Science and Engineering, Southern Cross University, East Lismore, New South Wales, Australia; 2 CSIRO Environment, St Lucia, Queensland, Australia; Bigelow Laboratory for Ocean Sciences, UNITED STATES OF AMERICA

## Abstract

Globally, multiple techniques are being trialed to accelerate ecosystem recovery in shallow coastal habitats. One technique aims to boost propagule supply in areas where the current supply is insufficient to re-establish depleted populations to ecologically significant levels. However, transferring large quantities of propagules requires large-scale methods to be tested to ensure safe and efficient collection and deployment into degraded habitats. This study aims to determine if the mass transfer of coral larvae through pumping techniques affects their survival, locomotion and settlement potential. A pilot experiment tested the effects of pumping at two flow rates, and a gravity-fed control, on *Acropora* cf. *tenuis* larval survival and settlement across four larval ages. Following pumping at days 2–5 following spawning, larval mortality rates were typically low (~0.8% and ~3% for low and high flow, respectively) and settlement rates similar. A subsequent experiment then investigated the effects of pumping on a mixed larval assemblage following *in situ* wild spawn slick collection and cultivation. Findings were similar to those of the pilot, confirming low average mortality rates (0.6–1%) with highest mortality (1.3%) for 3-day old larvae. At larval ages of 4-, 5- and 6-days post-spawning, pumping had no significant effects on locomotion abilities or settlement among larvae that were retrieved after transfer. For 3-day old larvae, locomotion following high flow pumping was marginally compromised, especially in the first minute following pumping, but locomotion increased significantly 5–10 minutes later. Larvae that were 3-days old and subjected to pumping (low or high) exhibited approximately 50% lower settlement rates compared to the control group. In contrast, no effects on settlement due to pumping were observed for 4-, 5-, or 6-day old larvae. Larval transfer, regardless of the technique employed, resulted in losses ranging from 21–27%. Losses were likely caused by some larvae becoming trapped in the fine mesh filter nets after transfer or lost through the aquarium system, with the extent of loss varying with the developmental stage of the larvae. Overall, results highlight that pumping coral larvae with a diaphragm pump can effectively facilitate their mass transfer when conducted from 4-days post-spawning onwards, at which point larvae are fully developed, motile and acquiring competency. Pumping techniques can therefore be utilized to facilitate increasing the rates and scales of larval restoration operations.

## Introduction

Efforts to stimulate ecosystem recovery in coastal marine environments globally are being trialed using diverse techniques at varying spatial scales. These efforts aim to increase propagule supply in regions where natural supply is insufficient to support the reestablishment of populations to ecologically significant levels [1–3]. On coral reefs, successful larval-based restoration trials at smaller scales [3,4] have shown strong potential for larger-scale application towards restoration of coral communities on degraded and recruitment-limited reefs [5,6]. Larval-based restoration methods enhance larval recruitment by supplying large numbers of sexually-derived coral larvae to targeted degraded reef sites [6–9]. Hundreds of millions of sexually-derived larvae can be collected following large-scale spawning events (‘wild coral spawn’), and can be reared *in situ* in floating pools [3,6,8–10] or *ex situ* in aquaculture-like facilities before being released onto target reef plots [4,8,11,12]. Enhancing the supply of competent larvae used in reseeding trials has resulted in initial coral settlement rates that exceed background natural recruitment in multiple trials [3,4,6,10].

Coral larval restoration typically follows five operational steps [8,9,13]; i) gravid coral checks ii) spawn collection, iii) transfer of gametes and embryos for mass larval culture (*in situ* or *ex situ*), iv) larval release onto target sites, and v) monitoring of initial larval settlement and survivorship through time [3,4,6,13,14]. Optimizations at each key phase are required to upscale coral larval restoration efforts. Trials to optimize the collection of wild coral spawn at scale have been conducted involving the harvesting of coral gametes and developing embryos from wild coral spawn slicks using various methods including floating spawn catchers [3,8] and industrial scale pumps [11]. A pilot study conducted around Heron Island (Great Barrier Reef [11]) resulted in a large-scale collection and cultivation of wild caught spawn from diverse species assemblages, with 29 million coral embryos reared for 5-days onboard a vessel with a 50,000 L aquaculture facility. That study highlighted the potential to transport large quantities of coral larvae from a collection site to a recruitment-limited receiving site, which would allow for transit over hundreds or thousands of kilometres during the larval rearing period [11]. Alternatively, mass culturing using *in situ* culture pools with nets is a low risk and cost efficient technique that minimizes transportation and infrastructure expense if large-scale translocation is not required [3,8]. However, both methods require the mass transfer of larvae during both collection and deployment. Improving how large quantities of coral larvae are concentrated and transferred onto degraded reefs requires the introduction of novel techniques to optimise larval restoration [5].

Minimising the time it takes to transfer larvae from culture onto targeted reef sites requires targeted research and development in order to i) manage differing settlement competencies of a mixed species larval cohort [15] collected from wild spawn slicks, ensuring larvae do not settle in culture prior to deployment; ii) reduce field operations costs (i.e., salaries, daily equipment hire) [4,6,13] and, iii) maximise settlement on reefs during optimal environmental conditions that reduce larval dispersal (i.e., slack current conditions) [16,17]. As culture densities often exceed what is ecologically necessary for a single target restoration site, methods also need to be considered in the latter phase of larval development to divide cultures for distribution across multiple reefs away from culture locations. One method includes the use of industrial pumps to transfer from cultures to damaged reefs [11]. However, pumping well-developed developed larvae during their settlement competency window (3-days and older) has only been trialed once [11], with mechanistic trials required to fully investigate the effects on larvae mortality, locomotion and settlement.

Pumping systems involve pressure fluctuations, shear stresses, and flow [18]. Various stress intensities associated with these systems, alongside a high threshold limit value, may cause expansion, rotation, deformation, and/or mortality to organisms being transferred within pumping systems. As coral embryos and larvae do not have a protective outer layer [19], their susceptibility to damage from shear forces and ease of fragmentation in the early phase of embryonic development is high [20]. Previous research has highlighted the susceptibility of coral embryos to these mechanisms, particularly the ease of fragmentation during the early phase of embryo development when embryo’s are in the 8–16 cell stage [11,20,21]. For example, wind speeds capable of generating small white caps at sea occur during some coral spawning events and create shear forces and turbulence on the ocean surface [21,22]. Experimental studies mimicking these condition have confirmed that whilst fragmentation increases to produce smaller coral embryo clones, fragmented embryos are able to develop into competent larvae [11,20]. Doropoulos et al. [11] found high survivorship following pumping despite high fragmentation of coral embryos. In both studies, the smaller fragmented embryos were capable of developing into competent larvae [11,20]. In contrast, pumping stress has been well documented in bony fish propagules, where the presence of a skeleton during embryonic development increases susceptibility to mechanical damage, resulting in growth deformities and other developmental issues [18,23].

When considering pumping during the later phase of coral larval development, actively swimming coral larvae require energy to function (locomotion and settlement), with the endogenous lipid energy reserves influencing larval longevity and maximum pelagic larval duration [24,25]. The effects of short-term exposure to pressure fluctuations, shear stresses, and flow accelerations from industrial pumping on actively swimming larvae remain unknown, and whether energy expenditure and stress following pumping compromises locomotive abilities and/or settlement also requires investigation. Therefore, this study aimed to determine the extent to which short-term exposure to pumping affects the survival, locomotive abilities and settlement potential of coral larvae across the early larval competency period. Specifically, we investigated: a) the immediate effects of pumping stress on larvae, including survival and locomotion; b) the impact of pumping on the timing of settlement (i.e., attachment and metamorphosis) of coral larvae; and c) the pumping flow rates and larval ages (3–6 days old) that promote the highest survivorship and settlement rates. The results aim to inform the potential for large-scale pumping methods to facilitate the efficient and safe transfer of ecologically relevant quantities of coral larvae to support large-scale larval restoration efforts.

## Methods & materials

Two experiments were conducted to determine the effects of pumping incurred under two flow rates (low = 0.7 L/sec; high = 1.6 L/sec) on coral larval survival, development and settlement capabilities. The first pilot experiment (herein referred to as experiment 1) was conducted at the Australian Institute of Marine Science’s National Sea Simulator (SeaSim;Townsville, Australia) using larvae from a single coral species. Gravid colonies of *Acropora* cf. *tenuis* were collected from Palm Island reefs (18.7345^o^ S, 146.5798^o^ E) and this species was chosen due to its abundance on inshore reefs in the central Great Barrier Reef (GBR). Colonies were transferred to outdoor flow-through holding tanks at SeaSim and maintained at 27^o^C under 30% shaded natural sunlight until spawning occurred on the 13^th^ of November 2022, 5-nights after the full moon. Following spawning, gamete bundles from numerous coral colonies were skimmed from the surface, and a 60 $\begin{array}{c} \mu \end{array}$m mesh sieve was used to separate eggs and sperm. Eggs were transferred to a separate 60L holding tank filled with 1 $\begin{array}{c} \mu \end{array}$m filtered seawater. Sperm were added at a concentration of 1 x 10^6^ sperm/ml to optimise fertilisation rates [26]. Once embryo cleavage was observed (~2 hours), embryos were rinsed to remove excess sperm [26] and transferred to a 500L culture tank where they remained for the duration of this experiment.

Experiment 2 was conducted at the Orpheus Island Research Station (OIRS) using a mixed-species cohort of wild caught larvae. Coral gametes and developing embryos were collected *in situ* from the reefs surrounding Orpheus Island (18.6161^o^ S, 146.4972^o^ E) following coral spawning on the 2^nd^ November 2023, 5-nights after the full moon. *Acropora* spp., *Montipora* spp., and *Porites* spp. are the dominant hard coral genera in the area [53], with embryos collected from slick formations occurring in this study likely comprised of a mixture of these genera. Samples of coral spawn slicks were collected using small surface skimmers at nearby reef locations and transferred into a 4 x 4 m floating larval pool with a 3 x 3m culture net for larval culture [6,8].

**Fig 1:**
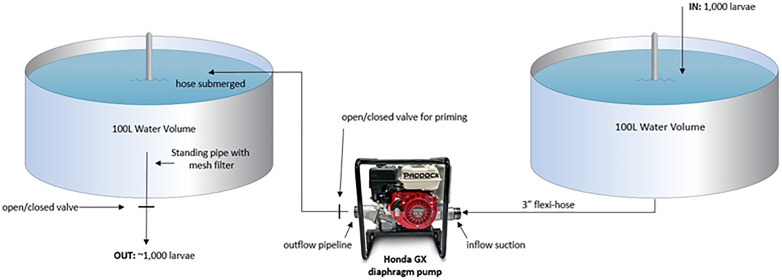
Experimental design used for larval pumping of experiments 1 and 2.

A Honda GX series diaphragm pump was used for experiments 1 and 2 due to its highest ranking for pumping slicks and embryos previously assessed using four practical criteria; pump priming, availability, handling and scalability [11]. The pump was aluminium, petrol driven, 45 kg in weight, and supported by a commercial 4-stroke engine. The selected diaphragm pump uses one-way 3-inch valves on the suction and discharge, in combination with a diaphragm, which rises and falls to draw in and discharge fluid (**Fig 1**).

### Experimental design

#### Pumping of coral larvae.

Experiment 1 (pilot): Acropora cf. tenuis larvae: Pumping trials started 2-days after spawning and then repeated daily from days 3–5. A total of 1,000 larvae were transferred from one tank to another using three different approaches; low flow pumping, high flow pumping, and a gravity-fed approach (i.e., control). In this pilot experiment, only one replicate of 1,000 larvae per day per treatment was able to be completed due to limited availability of larvae.

Prior to each experimental trial, 1,000 larvae were concentrated and counted under a microscope before being gently released into a 500 L holding tank containing 100L of seawater (including the seawater volumes required for pump priming and filling the inflow hose). Simultaneously to turning the pump on, the outflow valve was opened to allow the contents of the holding tank to be passed through the diaphragm and into a 500 L rounded outflow tank filled with seawater via a 3” flexible hose. This hose was submerged in the outflow tank to avoid potential shock associated with larvae hitting the water surface, and the rounded tank allowed the water from the hose to create an eddy which prevented collision of larvae with the tank walls. A ~ 1.5 m plankton mesh net (50 $\begin{array}{c} \mu \end{array}$m) was attached to the end of the outflow flexi-hose to capture larvae passing through the diaphragm. This process was repeated at two flow rates (low; 0.7 L/sec, high: 1.6 L/sec), which coincided with the lowest and highest settings of the pump. To determine the number of larvae trapped inside the pump after each trial, 50 L of seawater was flushed through the pump and collected in a separate plankton net. To compare the pumping approach with a passive (gravity-fed) transfer method, 1,000 *A*. *tenuis* larvae were gently poured through a funnel attached to the outflow hose into the same outflow tank and captured via a plankton mesh net.

Experiment 2: Mixed species larval assemblage: Pumping trials with larvae from wild spawn slick collection at OIRS began 3-days post-spawning once larvae exhibited signs of locomotion, and pumping trials were repeated at 4-, 5-, and 6-days post-spawning. Prior to experimental trials, larvae were collected from a 4 x 4 m *in situ* larval culture pool [9] and transferred to OIRS where larvae were counted and assessed for body shape or extent of deformation using a stereomicroscope and illuminated with cool LED light sources. Experiment 2 utilized the same treatments and flow rates as per experiment 1, however replication was increased to three trials per treatment per day. Prior to pumping both the inflow and outflow culture tanks were filled with 100 L of filtered seawater. The 100 L of the inflow tank included seawater required for pump priming and the inflow hose volume, with the outflow tank containing enough water for the hose to be submerged to avoid larval immersion disturbance associated with hitting the water surface. Once the pump was turned on, 1,000 larvae were added to the inflow culture tank where they were pumped via the hose through the diaphragm and released into the outflow tank. Once larvae were released into the outflow tank the water volume was lowered to 15 L with a 50 µm mesh filter placed within the tank’s outlet pipe to ensure larvae were not lost from the tank during this process. Once the water volume was lowered, the mesh filter was removed to allow the remaining water (containing larvae) to be released into a 25 L container. From there, larvae were gently scooped and concentrated until all larvae could be transferred into 50 mL falcon collection tubes for subsequent monitoring. As all larvae were counted, those missing from the initial 1,000 larvae sample after pumping and gravity transfer were classified as ‘unaccounted’.

### Larval survival & settlement assays

Following larval transfers from experiments 1 and 2, survival outcomes were determined as alive or dead. Larvae classified as alive were morphologically unaffected, had no lacerations or obvious injuries, and were capable of swimming and undergoing metamorphosis. Larvae classified as dead were initially identified due to deformations of the body plan, and showed no signs of movement. To confirm this status, all deformed and non-moving larvae were pipetted into a 6-well plate (1 larvae per well) comprised of ~18mL of seawater per well to monitor their survival (i.e., whether they regained movement or were able to metamorphose) or subsequent larval disintegration over 24-hours.

Experiment 1: Acropora cf. tenuis larvae: Larvae concentrated within the 50 mL falcon tubes were pipetted into Bogorov plankton counting trays and analysed for any deformations to their body plan under a stereo-microscope. Of the larvae showing signs of instantaneous survival (no signs of larval deformation, and active swimming), 120 larvae were pipetted across six cell culture wells (20 larvae per well) containing 1 cm^2^ *Porolithon* spp*.* (crustose coralline algae [CCA]) chips and filtered seawater (~16mL maximum volume per well), with another 120 larvae pipetted across six cell culture wells (20 larvae per well) without CCA. Wells were kept in a temperature controlled room (26^o^C), and assessed for settlement after both 24 h and 48 h.

Experiment 2: Mixed Larval Culture: After larval transfers via pumping or gravity transfers, 120 visibly unaffected larvae (no lacerations and larvae were swimming) were pipetted across six cell culture wells (20 larvae per well) containing 1 cm^2^ CCA chips of *Porolithon* spp*.* and filtered seawater, with another 120 larvae pipetted across six cell culture wells (20 larvae per well) without CCA. Larvae were assessed for settlement every 24h within a 7-day competency period (culture age day 3: 24h, 48h, 72h, 96h; culture age day 4: 24h, 48h, 72h; culture age day 5: 24h, 48h; culture age day 6: 24h). Water changes were conducted daily, whereby 50% of the total volume was replaced with new filtered seawater. Lids were kept on to prevent evaporation. Settlement assays were conducted in a temperature-controlled (26.5^o^C) laboratory with 12:12hr day/night light control.

### Larval locomotion

To determine if pumping had an effect on larval movement, locomotion assays were conducted in Experiment 2. A subsample of larvae that had been transferred into the 25 L container were gently scooped from the tub and transferred into a 6-well plate where the effects of each treatment on larval locomotion was recorded using a Canon G7x Mark III camera. Between 1–10 larvae were present within each well to monitor for locomotion. Recording began 5 minutes post-treatment and lasted for 20 minutes. Locomotion footage was analysed by determining the number of larvae capable of swimming after viewing footage for 20 minutes across five intervals. These intervals were defined as: 0–1 minute, 1–5 minutes, 5–10 minutes, 10–15 minutes, and 15–20 minutes post larval retrieval after pumping and gravity transfer. Videos with wells comprised of more than one larvae had to be monitored repeatedly, with frame by frame movements monitored across the monitoring intervals.

### Statistical analysis

All statistical analyses were completed in R (R Core Team 2022) and visualized using the “ggplot2” package (Wickham 2016). For all analyses, where model terms were significant, least-squares (marginal) means calculated with a Tukey adjustment were estimated using ‘emmeans’ to examine pairwise differences in settlement. Model diagnostics were checked using the “DHARMa” package [27] and assumptions validated.

*Larval Survival:* To investigate the effects of pumping on larval survival, the proportion of surviving larvae from experiment 1 (single species *A.* cf. *tenuis*) – larvae accounted for following transfers at final monitoring – was tested among pumping treatments (fixed effect; 3 levels) and larval age (fixed effect; 3 levels) using a Generalized Linear Model (GLM) with a binomial distribution using the package ‘glmmTMB’ [28]. The proportion of unaccounted larvae from the initial sample size (i.e., 1000 larvae) was tested using the same model.

To investigate the effects of pumping on larval survival from wild multispecies coral assemblages, the proportion of surviving larvae from experiment 2 (mixed species) was tested among pumping treatments (fixed effect; 3 levels), larval age (fixed effect; 3 levels), and their interaction, with transfer replicate included as a random effect, using a Generalized Linear Mixed Model (GLMM) with a binomial distribution using the package ‘glmmTMB’ [28]. The proportion of unaccounted larvae from the initial sample size (i.e., 1000 larvae) was tested among pumping treatments using the same model.

*Locomotion:* The proportion of “non-swimming” larvae from experiment 2 (mixed culture) was tested among treatments (fixed effect, 3 levels), larval age (fixed effect, 4 levels), time following the retrieval of larvae post-pumping (fixed effect, 4 levels), and their interaction using a GLM with a binomial distribution.

*Settlement:* To determine the effects of pumping on larval settlement competency following experiment 1 (*A.* cf. *tenuis*), the proportion of settled larvae within 24 h was tested among the additive effects of pumping treatment (fixed effect; 3 levels) and larval age (fixed effect, 4 levels) using a GLM with a binomial distribution. Following experiment 2 (mixed species larval culture), the proportion of larvae that settled within 24 h was tested among pumping treatment (fixed effect; 3 levels), larval age (fixed effect, 4 levels), and their interaction using a GLMM with a binomial distribution. Transfer replicate was included as a random effect.

## Results

### Larval survival outcomes

**Fig 2:**
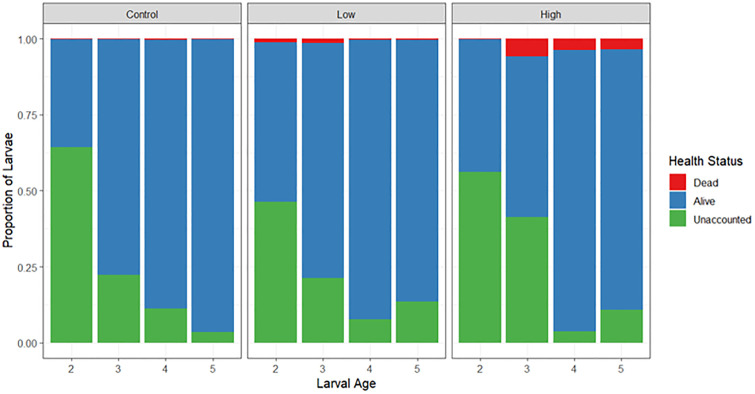
Survival outcomes (dead/alive/unaccounted) of *A.* cf. *tenuis* larvae following treatments (low flow, high flow, control) across four larval ages (2, 3, 4 and 5 days old). Note: this plot is presented as a stacked bar plot as there was one sample per larval stage per treatment level.

*Experiment 1: Effects of pumping on A. cf. tenuis larvae:* Overall, the majority of *A.* cf. *tenuis* larvae were largely unaffected by pumping, with survival averaging >95% across treatments. Whilst there were generally minor effects of pumping on larvae survival from 2–5 days post-spawning, larval age (p < 0.001) and pumping treatment (p < 0.001) significantly influenced survival outcomes (S1 Table, Fig 2). Larvae that passed through the diaphragm at a low and high flow rate experienced significantly higher mortality rates than the gravity-fed control (p < 0.01). Additionally, the high flow rate caused mortality rates that were significantly higher than the low flow rate (p < 0.001). When analysing the effects of larval age, larvae aged 3-, 4- and 5-days post-spawning experienced significantly higher mortality than larvae aged 2-days post-spawning (significant effect, p < 0.001).

The proportion of larvae unaccounted at the final monitoring after transfer was influenced by the additive effects of treatment and larval age (significant effect, p < 0.001, S2 Table, Fig 2). Overall, the high flow (27%) and control group (26%) had a significantly higher proportion of unaccounted larvae than the low flow (21%) treatment. In addition, the proportion of unaccounted larvae declined with larval age, with larvae transferred on day 2 post-spawning having a significantly higher proportion (53%) of unaccounted larvae than day 5 post-spawning (8%).

**Fig 3:**
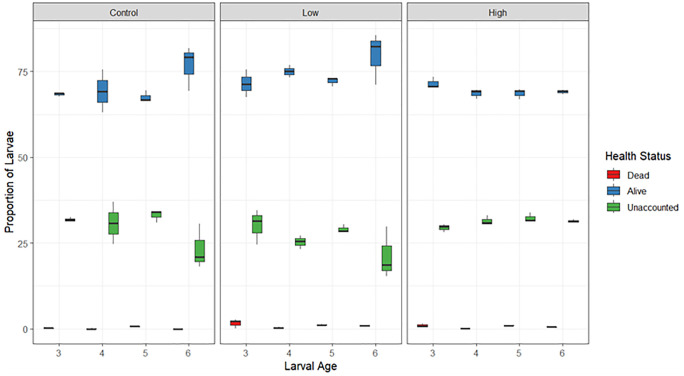
Boxplots of survival outcomes (alive/dead/unaccounted) from a mixed larval culture among treatments (low flow, high flow, control) across four larval ages (3, 4, 5 and 6 days old). Boxplot represents the interquartile range, which contains the middle 50% of the data from the 3 replicate samples per larvae stage per treatment level. The middle line shows the median.

*Experiment 2: Effects of pumping on a mixed larval assemblage from wild spawn slicks:* Overall, survival outcomes showed that larvae cultured from wild spawn slicks were mostly unaffected by pumping, with marginal mortality outcomes (low: ~ 1%; high: ~ 0.6%; control: ~ 0.2%). Whilst mortality in the study was marginal, a significant interaction between treatment and larval age was found to affect the proportion of surviving larvae (significant interaction, p < 0.001, S3 Table, Fig 3). More specifically, larvae that passed through the diaphragm pump at either low or high flow at 3-days post-spawning had significantly higher mortality rates than larvae in the gravity-fed control group. However, the mortality following pumping (low or high flow) was lowest when pumped at a larval age of 4-days post-spawning. No significant difference in mortality was found between larvae aged 5- or 6-days post-spawning among the pumping and gravity transfer treatments.

The proportion of unaccounted larvae following transfers was influenced by the interaction between treatment and larval age (significant interaction, p < 0.001, S4 Table, Fig 3). The control and high flow treatment resulted in a higher proportion of unaccounted larvae than the low flow treatment across 4- and 5-day old larvae. However, in 6-day old larvae, the high flow treatment recorded a higher proportion of unaccounted larvae (30%) than both the control (23%) and low flow (19%) treatment.

### Locomotion

**Fig 4:**
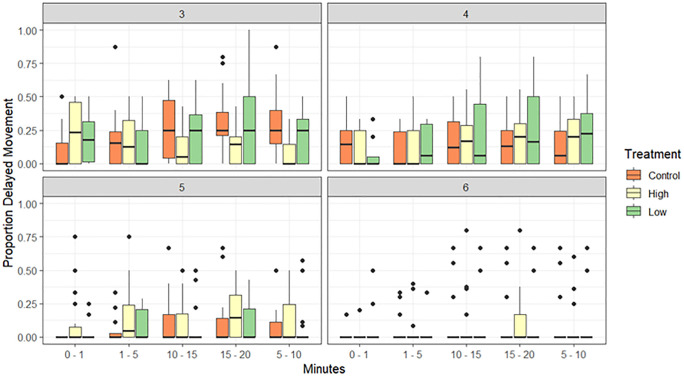
Proportion of “non-swimming” larvae at 5-minute intervals following treatments (low flow, high flow, control) in 3-, 4-, 5-, and 6-day old larvae.

*Experiment 2: Mixed larval culture:* When analysing the effects of delayed larval movement following pumping treatments, a significant interaction among time (minutes following pumping), larval age, and treatment was found (p < 0.01, S5 Table, Fig 4). At 3-days post-spawning, larvae pumped under the high flow treatment had a significantly higher proportion of non-swimming larvae within the first minute after transfer into the well-plates (~23%), when compared to the control (~9%). However, after 5–10 mins, larval movement in the high flow treatment increased, and the low flow treatment and control treatment larvae had significantly higher proportions of non-swimming larvae than the high pump treatment at all remaining timepoints (5–10 mins, 10–15 mins, 15–20 mins). No significant difference in the proportion of swimmers within time points or between treatments was found at larval ages 4- (83%), 5- (90%) or 6-days (94%) post-spawning.

### Settlement

*Experiment 1: A. cf. tenuis:* When analysing the proportion of *A.* cf. *tenuis* larvae capable of settling within 24-hours following pumping, settlement was only recorded at larval ages 4- and 5-days post-spawning across all treatments. Larval age was found to significantly influence settlement, whereby larvae aged 5-days post-spawning settled within 24-hours at a significantly higher rate (24%) compared to larvae aged 4-days post-spawning (14%, p < 0.001, S6 Table). No effect of pumping or gravity-fed larval transfer treatments was found, where settlement rates averaged 17% and 22%, respectively.

**Fig 5:**
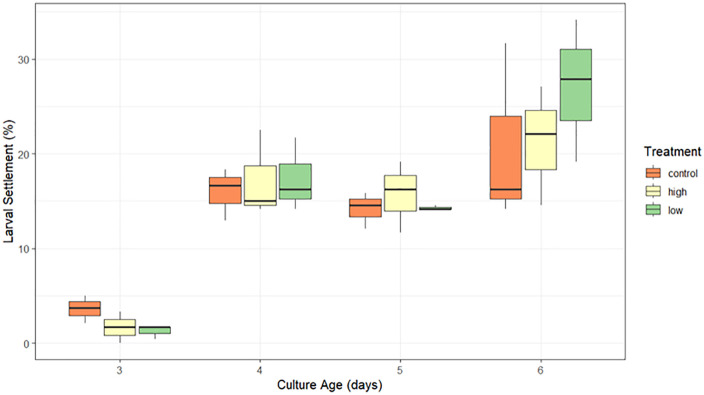
Proportion of larvae from a mixed larval assemblage capable of settling within 24h following treatments (low flow, high flow, control) across four culture ages (3, 4, 5 and 6 days old).

*Experiment 2: Mixed larval culture:* A significant interaction between treatment and larval age affected the proportion of larvae capable of settling within a 24-hour window from the assemblage of wild coral larvae, but only for 3-day old larvae (significant interaction, p < 0.02, S7 Table, Fig 5). Specifically, larvae pumped under a low flow rate on culture day 3 had a significantly lower 24-hour settlement rate than the control (p < 0.01, S5 Table, low: 1.3%, high: 1.7%, control: 3.6%). In contrast, no significant differences in settlement rates were identified between treatments on culture days 4, 5 or 6. Settlement was highest for 6-day old larvae (low: 27%, high: 21%, control: 21%).

## Discussion

Testing methods to upscale larval-based coral restoration practices has become increasingly important as the scale of coral loss increases globally [29–31]. As the collection and culturing of wild spawn slicks following coral spawning events has become a routine procedure capable of producing many millions of competent larvae [3,10,11], trialling methods that support the large-scale transfer of viable coral larvae onto degraded reefs that are i) time efficient, ii) low-cost, iii) low risk to larval survival, and iv) support spatial translocation across degraded reefs, is important for rapidly restoring coral cover and breeding corals to ecologically significant levels [5,6]. This study tested the ability of coral larvae to withstand the shear forces and turbulence generated by a diaphragm pump under two flow rates. Results from this study show that the pumping of coral larvae is suitable for large-scale application if pumping is conducted at a minimum of 4-days post-spawning, and filtering through a fine mesh net is avoided. Under these conditions, the technique has negligible effects on larval mortality, locomotion, or settlement among larvae that were retrieved after larval transfers.

### Overall larval survival outcomes

When testing for overall larval survival outcomes as a result of the shear forces and turbulence generated by pumping under two flow rates, larvae that were collected after transfers during the *A.* cf. *tenuis* and mixed wild larval culture trials remained largely unaffected. In *A.* cf. *tenuis*, survival differed significantly between pumping treatments, with the high flow rate causing slightly higher mortality (3.3%) than the low flow rate (0.7%). When pumping larvae from a mixed wild spawn larval culture, overall mortality among larvae that were retrieved after transfers was ~ 1% irrespective of the rate of flow in which larvae were passed through the diaphragm. Undocumented stressors likely occurring within the pipeline may have affected individual larvae, contributing to the minor mortality outcomes recorded in this study among larvae that were retrieved after transfer. It is possible that the presence of larvae changed the flow regime within the pipes, as the addition of particles can promote transition from a laminar to a turbulent flow [32]. Studies have highlighted further complexity to this dynamic [33], as the introduction of particles can induce local disturbances, which lead to a non-smooth flow even within the laminar regime. However, the influence ultimately depends upon the pipe to particle diameter ratios and concentration [33] and are therefore considered to be less likely to have been occurring in our study system. Secondly, if the particle doesn’t follow the streamlines of the water, the impact following particle collision with solid surfaces would likely be fatal [18]. A bend in the pipeline can create strain in the flow, which was present in this study. As the density of larvae utilized in this study is low, future studies may be needed to confirm density-dependent shearing risk.

It must be highlighted that mortality was also recorded in the gravity-fed control group, indicating that 0.2% of mortality outcomes occurred as a result of the mesh filtering process required to concentrate larvae into a significantly reduced water volume for monitoring. In addition, survival was found to be influenced by larval age in both *A.* cf. *tenuis* and mixed culture, with larvae aged 3-days post-spawning having a significantly lower survival. Coral larval longevity is dependent on their high lipid content [34–36]. The depletion of their lipid stores is typically associated with their loss of buoyancy [35,36], and may begin to change their vertical position in the water column either by passive sinking or active swimming [25,37]. As the timing of this study corresponded to the onset of larval locomotion and early settlement competency, inconsistencies in larval buoyancy within the pipeline may have generated local turbulence and/or increased the rate of larval collision with the wall of the pipeline. However, as the underlying effects of larval age and increased mortality within the pipeline are not yet fully understood, and given that mortality rates of larvae that were recovered after transfers were extremely low, the application of the pumping transfer technique is therefore recommended when larvae have been left to develop for at least 4-days.

The proportion of unaccounted larvae following Experiments 1 and 2 were relatively high irrespective of the technique employed (pumping or gravity-fed), with the average loss ranging from 21–27%. Approximately 25% of larvae were lost following gravity-fed transfers, indicating that the mesh filtering process was likely a large contributor towards this loss. As the onset of larval competency coincides with the development of secretory cells, larvae become capable of sampling and adhering to substrate [38,39]. Larval loss during the mesh filtering process may be related to the “sticky” property of the secretory cells that may cause some larvae to become stuck on the mesh prior to monitoring. Similarly, loss of larvae that can occur when the nets from *in situ* larval pools are lifted to concentrate larvae prior to transfer [8]. Transfer methods that involve filtering larvae may need to be modified to ensure that fewer larvae are lost from cultures, particularly if the stock of cultured larvae is limited. Larval loss was similar to gravity-fed transfers following pumping (20–30%), and this may be influenced by the secretory cells causing larvae to become stuck within the diaphragm as well as in the mesh filtering process for collecting larvae prior to monitoring. As the larval densities used in this study were orders of magnitude lower than those typically observed in concentrated spawn slicks following natural coral spawning events [13], demonstrating the feasibility of the pumping transfer at reduced densities, future work must explicitly test this approach under natural spawning densities to fully evaluate its ecological relevance and restoration potential.

### Pumping effects on larval locomotion

The swimming capabilities of larvae immediately following pumping were assessed in the wild spawn slick cultured larvae. Similar to larval mortality, while the effect size was negligible, the swimming capabilities of larvae aged 3-days was also significantly affected when passed through the diaphragm pump at a high flow rate. Here, a significantly higher proportion (92%) of three-day old larvae within the control group and larvae that passed through the diaphragm at a low flow rate began movement within 1 minute after transfer into the well-plates, compared with larvae from the high flow treatment (85%). However, larvae in the high flow treatment regained their swimming capabilities after 5–10 minutes and were capable of swimming for the remainder of the observation period. Whilst delayed swimming was still observed among some larvae after day 3, no significant differences among treatments were detected. In addition, by day 6, the impact on larval movement was minimal with only 0.06% of larvae that were tested exhibiting signs of delayed movement following pumping. Despite the small delays, large differences in transfer rates from larvae to settlement could potentially occur at target sites where larvae that are deployed for restoration due to initial drift with fast currents if deployments occur above the substrate [9,17].

Embryogenesis and larval development are the most energetically expensive periods for many marine invertebrates [40–42], including scleractinian coral larvae [24,43]. Despite this, some coral larvae have been recorded to complete metamorphosis and settlement beyond 100 days post-spawning [44–46], with survival estimates double that of non-metamorphosed larvae [24]. For larvae to be able to survive long periods in the plankton, they must possess a large supply of stored energy, have low metabolic rates, or be able to supplement their endogenous reserves through nutrient uptake or feeding [47,48]. A study recorded temporal changes in lipid content and respiration rates among four broadcast-spawning species in the first 3-weeks post-spawning [49], noting low respiration rates for eggs (<12h post-spawning) followed by a rapid increase during embryogenesis (12–36 h post-fertilization). This respiration peak coincides with high rates of cell division during embryogenesis, followed by the development of specialized cells associated with attachment and metamorphosis (36–96 h post-fertilization) [50]. Following this spike in respiration during development, respiration quickly falls to initial levels within 3–6 days [49]. Similarly, lipids are depleted rapidly during development and until metamorphosis at which point the rate of lipid utilization slowed dramatically [49]. The timing of the first larval transfer day via pumping was at 3-days post-spawning, which corresponds with the lowest performing locomotion assays following pumping under a high flow rate. These results suggest that pumping might temporarily disrupt ciliary coordination until normal movement was restored after 5–10 minutes. Following the 3^rd^ day post-spawning, larvae appeared to tolerate pumping flow rates better and may be more robust as they develop further and become competent and descend through the water column into benthic habitats where they have to cope with benthic currents and shear stress across a reefscape [17,52].

### Pumping effects on larval settlement

The capacity for larvae to survive and settle following pumping are essential requirements for the application of this transfer method in large-scale larval restoration endeavors. In *A.* cf. *tenuis*, no significant effects of pumping on larval settlement were found among larvae that were retrieved and tested for settlement responses. In the larvae reared from wild spawn slick samples, larvae exposed to high flow rates on the 3^rd^ day post-spawning showed a statistically significant reduction in settlement within 24hrs, however the effect size was minimal. This effect is likely influenced by larvae remaining precompetent at this age in a mixed assemblage [15]. Settlement rates did not differ significantly between treatments across the remaining larval ages that were tested (4-, 5- and 6-days post-spawning), and increased with larval age as expected, as competency of larvae from the mixed assemblage increased.

Utilising mixed species larvae collected from wild spawn slicks may create a challenge in identifying optimum periods for larval deployment or settlement onto devices due to differences among taxa in developmental rates and competency curves [15]. In this study, whilst settlement rates among larvae from the mixed species culture was highest on the 6th day post-spawning, this was the last day of census with only ~25% overall settlement. This suggests that a large proportion of the larvae were not yet competent to settle [45], highlighting the need to consider species-specific settlement windows when employing mass larval culturing to optimize success and minimize larvae losses [51]. Characterising the reproductive coral community prior to spawning, alongside the separate culturing of larvae collected between spawning nights, will provide insights into the minimum culturing period required to time larval transfers with peak settlement competency.

## Conclusion

The results of this study demonstrate that pumping of coral larvae through a diaphragm pump, irrespective of flow rate, is a low-risk technique for the transfer of coral larvae if pumping is conducted at a developmental phase where larvae are competent to settle (typically a minimum of 4-days post-spawning) and where the larvae are not filtered through fine mesh. Integrating industrial-scale larval culture and supply techniques will become increasingly important in managing routine production of ecologically significant numbers of coral larvae, and where scalability and translocation become key priorities to catalyze coral reef ecosystem recovery.

## Supporting information

S1 Table Proportion of dead *Acropora* cf. *tenuis* larvae distributed among treatments (low pump, high pump and control) and four larval ages (2, 3, 4 and 5 days post-spawning). (DOCX)

S2 Table Proportion of unaccounted *Acropora* cf. *tenuis* larvae distributed among treatments (low pump, high pump and control) and four larval ages (2-, 3-, 4- and 5-days post-spawning). (DOCX)

S3 Table Proportion of dead larvae distributed among treatments (low pump, high pump and control) and four larval ages from a mixed larval assemblage (3, 4, 5 and 6-days post-spawning). (DOCX)

S4 Table Proportion of unaccounted larvae distributed among treatments (low pump, high pump and control) and four larval ages from a mixed larval assemblage (3, 4, 5 and 6-days post-spawning). (DOCX)

S5 Table Proportion of non-swimming larvae between treatments (low pump, high pump and control), larval ages (3-, 4-, 5- and 6-days post-spawning) and minutes (0–1, 1–5, 5–10, 10–15, 15–20mins) from a mixed larval assemblage. (DOCX)

S6 Table Proportion of *Acropora* cf. *tenuis* larvae settling within 24 h between treatments (low pump, high pump and control) across larval ages (4- and 5-days post-spawning). (DOCX)

S7 Table Proportion of a mixed larval assemblage settling within 24 h between treatments (low pump, high pump and control) across larval ages (3, 4, 5 and 6-days post-spawning). (DOCX)
