# Supplementary material for: Evaluating pump-assisted larval transfer for scaling coral larval restoration interventions
Source: PLoS One. 2026 Apr 17;21(4):e0346728. doi: 10.1371/journal.pone.0346728 (PMC13089866; doi:10.1371/journal.pone.0346728)
Supplement: S1 Table — (DOCX) [file pone.0346728.s001.docx]

**Table S1.** Proportion of dead *Acropora* cf. *tenuis* larvae distributed among treatments (low pump, high pump and control) and four larval ages (2, 3, 4 and 5 days post-spawning).

| **Response (y) = Proportion** | **df** | **AIC** | **LRT** | **Pr(>Chi)** | **Pair-wise** |
| --- | --- | --- | --- | --- | --- |
| **Treatment (low pump, high pump, control)** | **2** | **237.86** | **1845.551** | **< 2.2e-16 ***** | Low, High > Control (<0.01)  High > Low (<0.0001) |
| **Larval Age** | **3** | **135.72** | **45.407** | **7.583e-10 ***** | Day 3, 4, 5 > 2 (p<0.0001) |
