# Supplementary material for: Evaluating pump-assisted larval transfer for scaling coral larval restoration interventions
Source: PLoS One. 2026 Apr 17;21(4):e0346728. doi: 10.1371/journal.pone.0346728 (PMC13089866; doi:10.1371/journal.pone.0346728)
Supplement: S2 Table — (DOCX) [file pone.0346728.s002.docx]

**Table S2**. Proportion of unaccounted *Acropora* cf. *tenuis* larvae distributed among treatments (low pump, high pump and control) and four larval ages (2-, 3-, 4- and 5-days post-spawning).

| **Response (y) = Proportion** | **df** | **AIC** | **LRT** | **Pr(>Chi)** | **Pair-wise** |
| --- | --- | --- | --- | --- | --- |
| **Treatment (low pump, high pump, control)** | **2** | **280.95** | **26.8** | **6.463e-10 ***** | High , Control > Low (p<0.0001) |
| **Larval Age** | **3** | **1539.02** | **1286.9** | **< 2.2e-16 ***** | Day 2 > 3, 4, 5 (p<0.0001)  Day 3 > 4, 5 (p<0.0001) |
