# Supplementary material for: Evaluating pump-assisted larval transfer for scaling coral larval restoration interventions
Source: PLoS One. 2026 Apr 17;21(4):e0346728. doi: 10.1371/journal.pone.0346728 (PMC13089866; doi:10.1371/journal.pone.0346728)
Supplement: S3 Table — (DOCX) [file pone.0346728.s003.docx]

**Table S3.** Proportion of dead larvae distributed among treatments (low pump, high pump and control) and four larval ages from a mixed larval assemblage (3, 4, 5 and 6-days post-spawning)

| **Response (y) = Proportion** | **df** | **AIC** | **LRT** | **Pr(>Chi)** | **Pair-wise** |
| --- | --- | --- | --- | --- | --- |
| **Treatment (low pump, high pump, control)** | **2** | **263.77** | **74.375** | **5.809e-15 ***** |  |
| **Larval Age** | **3** | **270.59** | **74.375** | **4.931e-16 ***** |  |
| **Treatment*Larval Age** | **6** | **202.21** | **26.843** | **0.0001549 ***** | **Culture Day 3:**  Low, High > Control (p<0.001)  **Culture Day 4:**  Control vs. High (p=0.99)  Control vs. Low (p=0.07)  High vs. Low (p=0.053)  **Culture Day 5:**  Control vs. High (p=0.88)  Control vs. Low (p=0.16)  High vs. Low (p=0.37)  **Culture Day 6:**  Control vs. High (p=0.4)  Control vs. Low (p=0.44)  High vs. Low (p=0.7)  **Low:**  Culture day 3, 5, 6 > 4 (p<0.05)  **High:**  Culture day 3, 5, 6 > 4 (p<0.05)  **Control:**  Culture day 5 > 3, 4 (p<0.01) |
