# Supplementary material for: Evaluating pump-assisted larval transfer for scaling coral larval restoration interventions
Source: PLoS One. 2026 Apr 17;21(4):e0346728. doi: 10.1371/journal.pone.0346728 (PMC13089866; doi:10.1371/journal.pone.0346728)
Supplement: S4 Table — (DOCX) [file pone.0346728.s004.docx]

**Table S4**. Proportion of unaccounted larvae distributed among treatments (low pump, high pump and control) and four larval ages from a mixed larval assemblage (3, 4, 5 and 6-days post-spawning)

| **Response (y) = Proportion** | **df** | **AIC** | **LRT** | **Pr(>Chi)** | **Pair-wise** |
| --- | --- | --- | --- | --- | --- |
| **Treatment (low pump, high pump, control)** | **2** | **441.74** | **39.810** | **2.157e-13 ***** |  |
| **Larval Age** | **3** | **455.08** | **55.153** | **2.048e-09 ***** |  |
| **Treatment*Larval Age** | **6** | **405.93** | **42.141** | **3.991e-07 ***** | **Culture Day 3:**  Control > High, Low (p<0.05)  High vs. Low (p=0.89)  **Culture Day 4:**  Control, High > Low (p<0.01)  Control = High (p=0.37)  **Culture Day 5:**  Control > Low (p<0.03)  Control = High (p=0.84)  **Culture Day 6:**  High > Control, Low (p<0.0001)  Control > Low (p<0.01)  **Low:**  Culture day 3, 4, 5 > 6 (p=0.0001)  **High:**  Culture day 3 vs 4 (p=0.81)  Culture day 3 vs 5 (p=0.14)  Culture day 3 vs 6 (p=0.28)  Culture day 4 vs 5 (p=0.59)  Culture day 4 vs 6 (p=0.80)  Culture day 5 vs 6 (p=0.98)  **Control:**  Culture day 3, 4, 5 > 6 (p=0.0001) |
