# Supplementary material for: Evaluating pump-assisted larval transfer for scaling coral larval restoration interventions
Source: PLoS One. 2026 Apr 17;21(4):e0346728. doi: 10.1371/journal.pone.0346728 (PMC13089866; doi:10.1371/journal.pone.0346728)
Supplement: S5 Table — (DOCX) [file pone.0346728.s005.docx]

**Table S5.** Proportion of non-swimming larvae between treatments (low pump, high pump and control), larval ages (3-, 4-, 5- and 6-days post-spawning) and minutes (0-1, 1-5, 5-10, 10-15, 15-20mins) from a mixed larval assemblage.

| **Response (y) = Proportion non-swimming larvae** | **df** | **AIC** | **LRT** | **Pr(>Chi)** | **Pair-wise** |
| --- | --- | --- | --- | --- | --- |
| **Treatment (low pump, high pump, control)** | **2** | **2162.5** | **0.564** | **0.7542** |  |
| **Culture day (3, 4, 5, 6)** | **3** | **2139.7** | **159.695** | < 2.2e-16 *** |  |
| **Minutes (0-1, 1-5, 5-10, 10-15, 15-20)** | **4** | **2196.1** | **38.135** | 1.051e-07 *** |  |
| **Treatment*Minutes** | **8** | **2148.3** | **15.172** | **0.05590** |  |
| **Treatment*Culture Day** | **6** | **2172.1** | **34.993** | 4.322e-06 *** |  |
| **Minutes*Culture Day** | **12** | **2150.8** | **25.599** | **0.01222** |  |
| **Treatment*Minutes*Culture Day** | **24** | **2149.2** | **41.561** | **0.01444** | **Culture Day 3**  0-1min  High > Control (p=0.0295)  Low vs. High (p=0.06.)  Low vs. Control (p=0.2)  **1-5min**  High vs. Low (p=0.27)  Low vs. Control (p=0.26)  Control vs. High (p=0.99)  **5-10min**  Low, Control > High (p<0.01)  **10-15min**  Low, Control > High (p<0.01)  **15-20min**  Low, Control > High (p<0.01)  **Culture Day 4**  0-1min  High vs. Low (p=0.82)  Low vs. Control (p=0.41)  Control vs. High (p=0.66)  **1-5min**  High vs. Low (p=0.91)  Low vs. Control (p=0.51)  Control vs. High (p=0.69)  **5-10min**  High vs. Low (p=0.39)  Low vs. Control (p=0.94)  Control vs. High (p=0.83)  **10-15min**  High vs. Low (p=0.99)  Low vs. Control (p=0.89)  Control vs. High (p=0.91)  **15-20min**  High vs. Low (p=0.91)  Low vs. Control (p=0.51)  Control vs. High (p=0.69)  **Culture Day 5**  **0-1min**  High vs. Low (p=0.13)  Low vs. Control (p=1.00)  Control vs. High (p=1.00)  **1-5min**  High vs. Low (p=0.39)  Low vs. Control (p=0.97)  Control vs. High (p=0.37)  **5-10min**  High vs. Low (p=0.66)  Low vs. Control (p=0.93)  Control vs. High (p=0.92)  **10-15min**  High vs. Low (p=0.39)  Low vs. Control (p=0.22)  Control vs. High (p=0.90)  **15-20min**  High vs. Low (p=0.30)  Low vs. Control (p=0.72)  Control vs. High (p=0.85)  **Culture Day 6**  0-1min  High vs. Low (p=0.44)  Low vs. Control (p=0.29)  Control vs. High (p=0.97)  **1-5min**  High vs. Low (p=0.29)  Low vs. Control (p=0.36)  Control vs. High (p=0.94)  **5-10min**  High vs. Low (p=0.39)  Low vs. Control (p=0.67)  Control vs. High (p=0.78)  **10-15min**  High vs. Low (p=0.19)  Low vs. Control (p=0.67)  Control vs. High (p=0.39)  **15-20min**  High vs. Low (p=0.15)  Low vs. Control (p=0.67)  Control vs. High (p=0.29) |
