# Supplementary material for: Evaluating pump-assisted larval transfer for scaling coral larval restoration interventions
Source: PLoS One. 2026 Apr 17;21(4):e0346728. doi: 10.1371/journal.pone.0346728 (PMC13089866; doi:10.1371/journal.pone.0346728)
Supplement: S6 Table — (DOCX) [file pone.0346728.s006.docx]

**Table S6.** Proportion of *Acropora* cf. *tenuis* larvae settling within 24 h between treatments (low pump, high pump and control) across larval ages (4- and 5-days post-spawning)

| **Response (y) = Proportion** | **df** | **AIC** | **LRT** | **Pr(>Chi)** | **Pair-wise** |
| --- | --- | --- | --- | --- | --- |
| Treatment (low pump, high pump, control) | 2 | 123.25 | 3.5239 | 0.1717096 |  |
| **Culture day (4 and 5)** | **1** | **135.65** | **13.9225** | **0.0001905 ***** | Culture day 5 > 4 (p<0.0002) |
