# Supplementary material for: Evaluating pump-assisted larval transfer for scaling coral larval restoration interventions
Source: PLoS One. 2026 Apr 17;21(4):e0346728. doi: 10.1371/journal.pone.0346728 (PMC13089866; doi:10.1371/journal.pone.0346728)
Supplement: S7 Table — (DOCX) [file pone.0346728.s007.docx]

**Table S7.** Proportion of a mixed larval assemblage settling within 24 h between treatments (low pump, high pump and control) across larval ages (3, 4, 5 and 6-days post-spawning)

| **Response (y) = Proportion** | **df** | **AIC** | **LRT** | **Pr(>Chi)** | **Pair-wise** |
| --- | --- | --- | --- | --- | --- |
| Treatment (low pump, high pump, control) | 2 | 262.29 | 1.43 | 0.4882 |  |
| **Culture day (3, 4, 5 and 6)** | **3** | **667.21** | **408.36** | **<2e-16 ***** |  |
| **Treatment*Culture Day** | **6** | **266.86** | **15.524** | **0.01655 *** | **Culture Day 3:**  Control > Low (p<0.01)  High = Low  High = control  **Culture Day 4:**  Control = Low = High  **Culture Day 5:**  Control = Low = High  **Culture Day 6:**  Control = Low = High  **High:**  Culture day 4, 5, 6 > 3 (p<0.0001)  **Low:**  Culture day 4, 5, 6 > 3 (p<0.0001)  Culture day 6 > 4, 5 (p<0.0001)  **Control:**  Culture day 4, 5, 6 > 3 (p<0.0001)  Culture day 6 > 5 (p<0.03) |
